# Development of a paradigm to investigate mechanisms of divided and selective attention impairment in the rodent models of Alzheimer’s disease

**DOI:** 10.1186/1750-1326-8-S1-P6

**Published:** 2013-09-13

**Authors:** Gytis Baranauskas, Natasa Svirskiene, Gytis Svirskis

**Affiliations:** 1 Neuroscience Institute, Lithuanian University of Health Sciences, Kaunas, Lithuania

## 

In Alzheimer’s disease impaired attention can be detected almost immediately after the first signs of memory loss and is considered to be a major cause of difficulties in the patients everyday life in the early stages of Alzheimer’s disease. Several types of attention are recognized such as divided, selective and sustained attention. Selective and divided attention are impaired most in the Alzheimer’s disease patients while sustained attention remains relatively intact. However, most attention tests in animal models of Alzheimer’s disease such as the five-choice serial reaction time task evaluates sustained but not other attention types. We decided to profit from a recent finding that Alzheimer’s patients have an impaired ability to detect objects approaching on a collision course in tests for selective and divided visual attention [1,2]. Collision detection is conserved across species and is performed by specialized collision-sensitive neurons in the superior colliculus of both in humans and rodents facilitating extension to humans of the results obtained in animals.

However, so far little is known about collision-sensitive neurons in rats and mice and such a visual stimulus has not been used in attention tasks in rodents. To establish a paradigm for selective and divided visual attention tests in rodents, we recorded collision sensitive neurons in the rat superior colliculus by employing tetrode extracellular recordings in male, >1 month old rats. Collision sensitive neurons in the superficial layers of the superior colliculus were identified by their ability to respond to stimuli imitating collision but not to the ones veering slightly off. Collision sensitive neurons responded well to both dark (a black image on a white background) and bright (a bright image on a dark background) stimuli. Most putative collision-sensitive neurons could be assigned to eta class. Importantly, responses of collision-sensitive neurons were highly dependent on contrast, a parameter to be used to evaluate divided attention by presenting two different collision stimuli of different contrast. We will use these tests to investigate the effects of a 2-week intracerebroventricular infusion of beta-amyloid-(1-42), a rat model of Alzheimer’s disease [3], on divided and selective attention. This work was supported by a Lithuanian Research Council grant Nr. VP1-3.1-SMM-08-K-01-022.
